# Saccadic eye movement abnormalities in autism spectrum disorder indicate dysfunctions in cerebellum and brainstem

**DOI:** 10.1186/2040-2392-5-47

**Published:** 2014-09-16

**Authors:** Lauren M Schmitt, Edwin H Cook, John A Sweeney, Matthew W Mosconi

**Affiliations:** Center for Autism and Developmental Disabilities, University of Texas Southwestern, 5323 Harry Hines Blvd, Dallas, TX 75390-9086 USA; Department of Psychiatry, University of Illinois at Chicago, 1747 W. Roosevelt Rd (MC 747), Chicago, IL 60608 USA; Centre for Autism Spectrum Disorders, Bond University, Gold Coast, QLD 4229 Australia; Departments of Psychiatry and Pediatrics, University of Texas Southwestern Medical Center, 5323 Harry Hines Blvd, Dallas, TX 75390-9086 USA

**Keywords:** Autism spectrum disorder (ASD), Sensorimotor, Eye movement, Saccade, Cerebellum, Brainstem

## Abstract

**Background:**

Individuals with autism spectrum disorder (ASD) show atypical scan paths during social interaction and when viewing faces, and recent evidence suggests that they also show abnormal saccadic eye movement dynamics and accuracy when viewing less complex and non-social stimuli. Eye movements are a uniquely promising target for studies of ASD as their spatial and temporal characteristics can be measured precisely and the brain circuits supporting them are well-defined. Control of saccade metrics is supported by discrete circuits within the cerebellum and brainstem - two brain regions implicated in magnetic resonance (MR) morphometry and histopathological studies of ASD. The functional integrity of these distinct brain systems can be examined by evaluating different parameters of visually-guided saccades.

**Methods:**

A total of 65 participants with ASD and 43 healthy controls, matched on age (between 6 and 44-years-old), gender and nonverbal IQ made saccades to peripheral targets. To examine the influence of attentional processes, blocked gap and overlap trials were presented. We examined saccade latency, accuracy and dynamics, as well as the trial-to-trial variability of participants’ performance.

**Results:**

Saccades of individuals with ASD were characterized by reduced accuracy, elevated variability in accuracy across trials, and reduced peak velocity and prolonged duration. In addition, their saccades took longer to accelerate to peak velocity, with no alteration in the duration of saccade deceleration. Gap/overlap effects on saccade latencies were similar across groups, suggesting that visual orienting and attention systems are relatively spared in ASD. Age-related changes did not differ across groups.

**Conclusions:**

Deficits precisely and consistently directing eye movements suggest impairment in the error-reducing function of the cerebellum in ASD. Atypical increases in the duration of movement acceleration combined with lower peak saccade velocities implicate pontine nuclei, specifically suggesting reduced excitatory activity in burst cells that drive saccades relative to inhibitory activity in omnipause cells that maintain stable fixation. Thus, our findings suggest that both cerebellar and brainstem abnormalities contribute to altered sensorimotor control in ASD.

**Electronic supplementary material:**

The online version of this article (doi:10.1186/2040-2392-5-47) contains supplementary material, which is available to authorized users.

## Background

Impaired social communication abilities and restricted, repetitive behaviors represent the defining characteristics of autism spectrum disorder (ASD) [1]. However, the majority of affected individuals also show sensorimotor abnormalities that may precede the emergence of social communication and cognitive deficits early in life [2–5]. Improved understanding of characteristic motor deficits could therefore facilitate early detection. It also could provide insight into the pathophysiological mechanisms of ASD, as the brain systems supporting sensorimotor control are better understood than those supporting many higher-level cognitive and social processes.

In additional to atypical visual scan patterns while looking at faces and during social interactions [6–8], individuals with ASD show abnormalities in their the sensorimotor control of eye movements when viewing simple visual stimuli (illuminated dots on a blackened screen) [9–11] and human faces [12]. When making rapid shifts in eye gaze (saccades), individuals with ASD show reduced accuracy [9, 13–15] and increased trial-to-trial variability of saccade accuracy [9, 13, 16]. These deficits appear to be more severe in children compared to adolescents with ASD, suggesting that the ability to precisely control eye movements may mature at a delayed rate [14]. Saccade abnormalities also are sometimes seen in unaffected family members of individuals with ASD [17], highlighting their promise as endophenotypes for parsing heterogeneity and tracking familial risk in this disorder.

Some studies of saccade dynamics in individuals with ASD have documented reduced peak velocity [15] and increased duration [12, 16], while others have not [9, 13, 14, 18, 19]. These inconsistencies may result from differences in subject samples and methodologies across studies. Studies utilizing small samples (n ≤15) may lack power to detect abnormalities that may be variably present across affected individuals [13, 15, 16, 19]. For instance, one study documented abnormality in saccade dynamics, particularly in peak velocity, in one participant with ASD despite no overall group differences [19]. Moreover, some studies examined endogenously-cued or intentional saccades [12, 14–16], and others examined exogenously-cued or reflexive visually-guided saccades to targets with unpredictable onset timing and spatial location [9, 11, 13, 18, 19]. Abnormalities in saccade dynamics have been noted consistently in tasks when subjects make endogenously-cued saccades [15, 16] which are under significant top-down control from the cerebral cortex [20]. Alternatively, studies of visually-guided saccades have had less consistent findings, perhaps due to testing only small saccades in some studies, as deficits appear greater in larger saccades [17, 18]. Only one study examined the acceleration and deceleration of visually-guided saccades, however, healthy control and ASD groups did not significantly differ [13]. These results may suggest that saccade dynamics are only affected when cortical brain structures are involved. However, it is also possible that the small sample sizes and saccade amplitudes examined in previous studies may have limited sensitivity to detect abnormalities within cerebellar and brainstem structures supporting these functions. Furthermore, in contrast to saccade accuracy, there is less evidence supporting developmental improvement in saccade dynamics in both healthy [21, 22] and ASD samples during childhood and adolescence [14]. However, one study examining healthy controls across lifespan demonstrated increased peak saccade velocity from early childhood to adolescence, and subsequent decline in late adulthood [23]. With few studies examining the developmental changes of saccade dynamics in individuals with ASD, and inconsistent findings in healthy populations, a better understanding of these possible age-related changes is needed.

Saccadic eye movements are controlled by highly specialized cortico-cerebellar-brainstem circuitry [24]. Saccade initiation occurs when pontine burst cells are simultaneously released from their tonic inhibition by omnipause cells and driven by excitatory signals from the superior colliculus [24, 25]. Thus, the process of reflexive saccade initiation requires a temporally synchronized excitation of burst cells and release of those burst cells from tonic inhibitory input. The reverse of this process terminates saccades. Dynamic characteristics of saccades including their duration, peak velocity, acceleration and deceleration depend on the firing rates of pontine burst cells in the context of their release from omnipause cell inhibition [26].

Medioposterior lobules VI to VII of the cerebellum and the caudal fastigial nuclei to which they project act to maintain saccade accuracy, compensating for any systematic error via feedforward input to the brainstem, permitting precise refocusing of the fovea onto objects of interest [26–29]. In addition, saccade error can be partially corrected online, primarily via lobules VI-VII of cerebellum, by reducing or extending the deceleration phase of saccades [30–33]. Thus, both pontine brainstem nuclei and the cerebellum regulate specific aspects of saccades that can be evaluated relatively independently in order to identify functional abnormalities in these two brain regions.

The brainstem and cerebellum have been implicated in postmortem and magnetic resonance imaging (MRI) studies of ASD. Structural MRI studies have demonstrated pontine alterations that might cause eye movement abnormalities [34–38]. Postmortem studies show reduced numbers of Purkinje cells in the cerebellar vermis, hemispheres and deep nuclei [36, 39–42], as well as reduced cell counts in the fastigial nuclei [43]. Structural MRI studies have documented reduced grey matter volume within the cerebellar cortex [44] and hypoplasia of the cerebellar vermis lobules VI -VII [45, 46]. Reduced activation in the cerebellum during saccades has been reported in a functional MRI study [11]. Our group and others have recently demonstrated compromised cerebellar-dependent saccade adaptation in ASD, which provides perhaps the most direct neurobehavioral evidence of dysfunction within the cerebellar vermis [47, 48].

Gap/overlap paradigms are useful for examining saccade latencies during conditions with different levels of fixations on a central cue [49, 50]. This has been shown to be sensitive to saccade abnormalities in infants at risk for ASD [4], and is likely to some degree to be influenced by factors associated with visual attention linked to visual fixation. However, the extent to which latency differences are due to attentional versus oculomotor processes remains a topic of debate [51–53]. Previous studies in ASD using gap/overlap paradigms have yielded mixed results, with one showing no abnormality in the overlap effect in participants with ASD [54], and the other showing a reduced overlap effect [55]. Thus, the level of alteration in visual attentional engagement in ASD remains equivocal.

The current study aimed to examine the accuracy and dynamics of reflexive saccadic eye movements in a relatively large sample of individuals with ASD across a broad age range in order to evaluate the functional integrity of cerebellar and brainstem circuitry supporting distinct sensorimotor components of saccade control. Our secondary aim was to examine the effect of manipulating attention-shifting processes on saccade latencies utilizing gap/overlap paradigms. We hypothesized that individuals with ASD would show deficits in saccade accuracy, as well as impairments in saccade dynamics, based upon anatomical findings in the cerebellar and brainstem systems that support these functions respectively. We also hypothesized that attentional deficits would contribute to saccade initiation abnormalities in ASD.

## Methods

### Participants

A total of 65 individuals with ASD and 43 healthy controls matched in age (between 6 and 44-years-old), gender ratio and nonverbal IQ were examined (Table 1). As the oculomotor task placed minimal verbal demands on participants, we matched the ASD and control groups on nonverbal IQ as opposed to verbal ability. We have used this approach previously [9–11, 47] in order to include a more representative sample of individuals with ASD who are known to have more severe verbal deficits compared to nonverbal deficits [56] and large discrepancies between verbal and nonverbal abilities [57, 58]. Participants with ASD were recruited from outpatient clinics at the University of Illinois at Chicago Medical Center and via flyers posted in the community. A diagnosis of ASD, including autistic disorder, Asperger’s syndrome or pervasive developmental disorder-not otherwise specified (PDD-NOS) was established using the Diagnostic and Statistical Manual of Mental Disorders, Fourth Edition, Text Revision (DSM-IV-TR) criteria, as confirmed by research reliably trained clinicians using the Autism Diagnostic Interview-Revised (ADI-R; [59]), the Autism Diagnostic Observation Schedule (ADOS; [60]) and by expert clinical opinion (EC, MM). The participants with ASD were excluded if they had a genetic disorder associated with ASD (such as Fragile X syndrome) or a medical history of non-febrile seizures.

**Table 1:** **Demographic characteristics for participants with ASD and healthy controls**

Demographic characteristic	Age group	ASD (N = 65)	Control (N = 43)
Age group (n, %)	6-11	26 (40)	15 (35)
	12-18	23 (35)	12 (28)
	19+	16 (25)	16 (37)
	Total	65	43
Age in years	6-11	9.4 (1.4)	9.0 (1.6)
	12-18	14.0 (1.8)	14.1 (2.0)
	19+	26.3 (7.4)	26.1 (5.1)
	Total	15.2 (7.7)	16.8 (8.3)
Gender (% male)	6-11	73	73
	12-18	87	67
	19+	88	81
	Total	82	74
Full scale IQ	6-11	100 (17)*	113 (15)
	12-18	94 (19)*	107 (15)
	19+	102 (18)	110 (8)
	Total	98 (18)**	110 (13)
Verbal IQ	6-11	99 (16)**	116 (19)
	12-18	93 (22)*	110 (16)
	19+	109 (22)	109 (13)
	Total	99 (21)	106 (12)
Nonverbal IQ	6-11	102 (18)	109 (16)
	12-18	99 (19)	98 (10)
	19+	100 (22)	109 (8)
	Total	100 (20)	106 (12)
ADOS Total†	6-11	12.6 (5.7)	
	12-18	10.7 (3.4)	
	19+	9.8 (4.1)	
	Total	11.2 (4.7)	
ADI social total	6-11	19.9 (5.8)	
	12-18	21.5 (4.9)	
	19+	17.8 (6.9)	
	Total	20.0 (5.8)	
ADI communication total	6-11	16.2 (4.6)	
	12-18	16.9 (4.4)	
	19+	13.3 (5.8)ª	
	Total	15.8 (4.9)	
ADI repetitive behavior total	6-11	6.5 (2.6)	
	12-18	5.9 (2.4)	
	19+	4.8 (2.0)	
	Total	5.9 (2.4)	

Means (SDs) are presented for age and IQ.

*P <0.05; ** *P* <0.01.

†ADOS totals are based on revised algorithms [61] except for those participants (n = 22) who completed Module 4.

ªParticipants with ASD in the 19 years of age and older age band have significantly lower ADI Communication Total scores than those in the 12 to 18-years-old age band (*P* = 0.040).

ASD, autism spectrum disorder; IQ, intelligence quotient; ADOS, Autism Diagnostic Observation Scale; ADI, Autism Diagnostic Interview-Revised.

Healthy community controls were recruited through newspaper advertisements. All had a score of less than or equal to 8 on the Social Communication Questionnaire [62]. Healthy controls were excluded if they reported a personal history of psychiatric illness, a first-degree relative with a major psychiatric illness (such as schizophrenia), or a first- or second-degree relative with ASD. No participants had a previous head injury resulting in loss of consciousness, were taking any medications known to affect oculomotor function including stimulants, anticonvulsants or antipsychotics [63], consumed caffeine within 24 hours of testing, used nicotine within one hour prior to testing or had corrected far visual acuity of less than 20/40. Individuals completed the Differential Ability Scales ([64]; N = 75; <18-years-old) or Wechsler Abbreviated Scale of Intelligence ([65]; N = 33; ≥18-years-old) to assess intellectual abilities. Individuals with a full-scale IQ score of under 80 were not included. Adult participants provided written consent and minors provided assent in addition to written consent from their legal guardian. This study was approved by the Institutional Review Board of the University of Illinois at Chicago (Protocol #2002-0286 and #2007-0239).

### Procedures

Participants were tested in a darkened black room and positioned in a chin-rest with forehead and occipital restraints to minimize head movement. They were seated 60 cm from a 40-inch anti-glare LCD screen monitor (Samsung SyncMaster 400P; Samsung, Seoul, Korea) with a resolution of 1024 × 768 and a 75 Hz refresh rate. Electrodes placed at the lateral and nasal canthi of each eye and above and below the left eye monitored saccadic eye movements and blinks, respectively, using direct current electro-oculography (EOG; Grass Neurodata 12 Acquisition System; Astro-Med, Inc, West Warwick, Rhode Island, United States). We elected to use EOG rather than infrared recording in order to assess larger amplitude saccades (20 to 30 degrees), which our group and others have shown to be more severely impaired in ASD [13, 17]. EOG signals were digitized at 500 Hz with a 12-bit A/D converter (DI-720 from Dataq Instruments, Akron, Ohio, United States). Visual stimuli subtending 0.5 degrees of visual angle were presented in the horizontal plane at eye level (Flash MX Actionscript 2; Adobe Systems, San Jose, California, United States). Digital finite impulse response filters with non-linear transition bands were applied with a gradual transition band (from pass to no pass) between 20 and 65 Hz for velocity and position data, and between 30 and 65 Hz for acceleration data. Data from each trial were visually inspected offline and scored without knowledge of participant characteristics. Trials were assessed to determine the presence of confounding measurements (such as excessive noise in the signal, large head movements or failed task compliance). If the scorer judged any of these factors to be present, the trial was omitted from analyses.

All participants completed a visually-guided saccade task consisting of one block of 30 gap trials and one block of 30 overlap trials. The order of gap and overlap blocks was counterbalanced across participants. Gap trials included a 200 ms temporal gap between central fixation offset and peripheral target onset. In overlap trials, the central fixation target remained present for 200 ms after peripheral target onset. Trials began with a central target appearing for 1.5 to 2.5 seconds (varied randomly in 200 ms intervals). Peripheral targets were presented for 1.5 seconds at unpredictable locations 10, 20 or 30 degrees to the right or left of center fixation. Participants were instructed to look towards the peripheral target when it appeared.

Trials were calibrated independently using fixation data of central and peripheral target locations. Each trial was manually calibrated by marking stable center fixation prior to trial onset, and at the target location after the participant acquired the peripheral target. If signal drift or head movement occurred during the performance of the task, trials were recalibrated using within-trial data from fixation of targets of interest as we have done previously [9, 17, 18, 66–69]. Saccade onset and offset were marked when eye velocity rose above and below 30 degrees per second, respectively. Saccades with latencies less than 70 ms were considered anticipatory and not included in analyses. Trials in which blinks occurred between 100 ms prior to stimulus presentation and the end of the primary saccade were not included.

**Figure 1:**
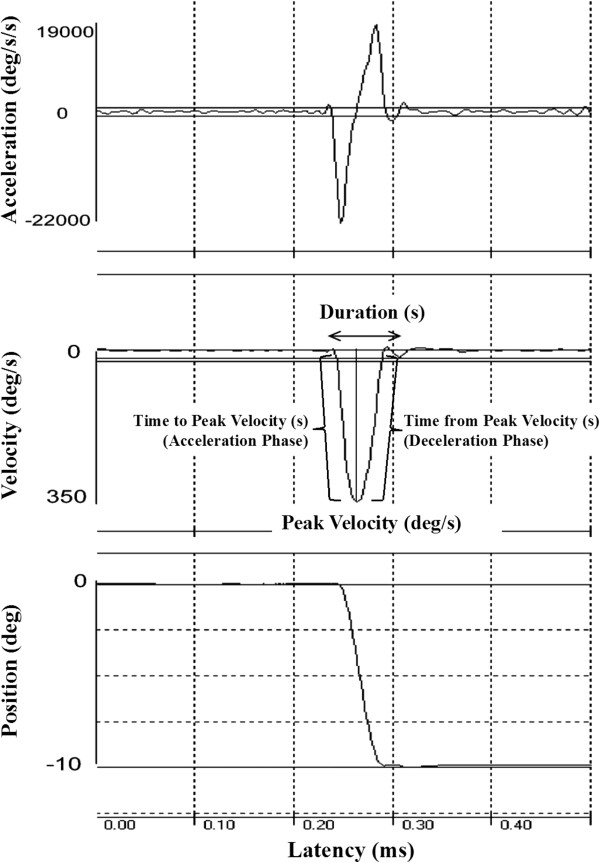
**A representative sample of position (bottom), velocity (middle) and acceleration/deceleration (top) traces of a single visually-guided saccade.** Parameters used to define the dynamics of each saccade, including the peak velocity and duration of the movement, and the acceleration and deceleration phases of the movement are shown in the velocity trace. Deg, degrees; s, seconds.

The latency, accuracy and dynamics were measured for each saccade (Figure 1). Latencies were calculated based on the difference between peripheral target onset and saccade initiation. Saccade accuracy was examined using the absolute value of the spatial error in degrees of primary saccades relative to peripheral target position. We measured the absolute value of spatial error between primary saccade amplitude and target location because we have previously observed both hypometric (target undershoot) and hypermetric (target overshoot) saccades in participants with ASD [9]. The mean number of saccades made to acquire the peripheral targets was computed for each individual.

The peak velocity and duration of each saccade were measured. As the velocity and duration of saccades increase with increasing saccade amplitudes according to a ‘main sequence’ [70], we also examined velocity:amplitude and duration:amplitude relationships. We compared the slopes of both the velocity:amplitude and duration:amplitude relationships to identify between-group differences. In addition, we measured peak acceleration, peak deceleration, the time from saccade onset to peak saccade velocity (duration of saccade acceleration) and the time from peak saccade velocity to the end of the saccade (duration of saccade deceleration). Lastly, we examined variability of saccade accuracy, velocity, duration, acceleration, deceleration, the durations of saccade acceleration and deceleration and latency by calculating their standard deviation across trials for each subject.

We used a series of 2 × 3 × 2 analysis of variance to examine the effects of diagnostic group (ASD versus control), target location (10° versus 20° versus 30°) and condition (gap versus overlap) on saccade performance. No effects of the direction of eye movements or interactions between saccade direction and diagnostic group were seen, therefore trials were collapsed across rightward and leftward target displacements. To examine saccade performance across development, we contrasted group differences at three different developmental stages (childhood: 6 to 11 years of age versus adolescence: 12 to 18 years of age versus adulthood: 19 and older), which were chosen based on our previous work investigating developmental trajectories of motor performance in ASD relative to healthy individuals [71].

## Results

### Latency

**Figure 2:**
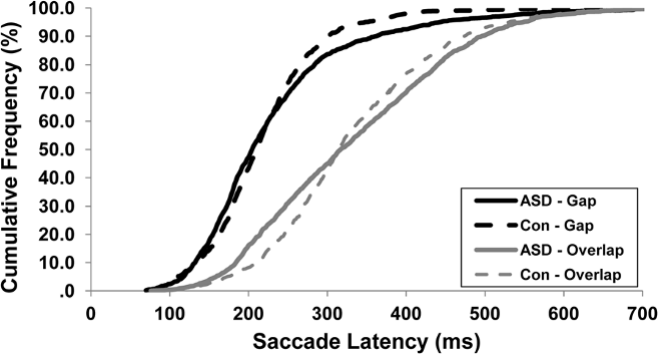
**Cumulative frequency plot of gap and overlap saccade latencies for participants with ASD and healthy controls.** Saccade latencies for overlap trials were significantly greater across participants. The extent to which latencies increased did not differ between groups, but latency variability was increased in ASD. ASD, autism spectrum disorder; Con, control.

As expected, the latencies of saccades during overlap trials were longer than those during gap trials (Table 2; F(1,104) = 297.81; *P* <0.001). The extent to which overlap trial saccades were delayed relative to gap trial saccades did not differ between groups (F(1,104) = 2.63; *P* = 0.108). No differences in mean saccade latencies were seen between participants with ASD and healthy controls (F(1,104) = 0.57; *P* = 0.454), however participants with ASD showed greater trial-to-trial variability in their saccade latencies (Figure 2; F(1,103) = 9.90; *P* = 0.002).

**Table 2:** **Saccade accuracy and latencies for participants with ASD and healthy controls**

	ASD	Control
**Saccade error (absolute value in degree of visual angle)**		
10 deg	1.46 (0.43)*	1.27 (0.07)
20 deg	2.40 (0.72)***	1.86 (0.11)
30 deg	3.33 (1.12)**	2.60 (0.17)
**Trial-wise variability (SD) of saccade error (absolute value in degrees)**		
10 deg	1.11 (0.37)	1.03 (0.37)
20 deg	1.90 (0.58)***	1.31 (0.58)
30 deg	2.79 (0.58)**	2.07 (0.29)
**Saccade amplitude in degrees**		
10 deg	9.78 (0.82)	9.69 (0.82)
20 deg	19.11 (1.28)	19.20 (1.28)
30 deg	27.71 (1.68)	28.14 (1.68)
**Trial-wise variability (SD) of saccade amplitude in degrees**		
10 deg	1.91 (0.64)	1.86 (0.65)
20 deg	2.75 (0.83)*	2.30 (0.83)
30 deg	3.86 (1.19)***	2.10 (1.19)
**Saccade latency in ms**		
10 deg	255 (52)	248 (52)
20 deg	275 (58)	273 (58)
30 deg	313 (55)	300 (55)
**Trial-wise variability (SD) of saccade latency in ms**		
10 deg	80 (30)**	64 (21)
20 deg	90 (37)**	66 (37)
30 deg	90 (32)*	75 (32)

Mean (SD).

*P <0.05; **P <0.01; *** *P* <0.001.

ASD, autism spectrum disorder; SD, standard deviation; ms, milliseconds; deg, degrees.

### Accuracy

**Figure 3:**
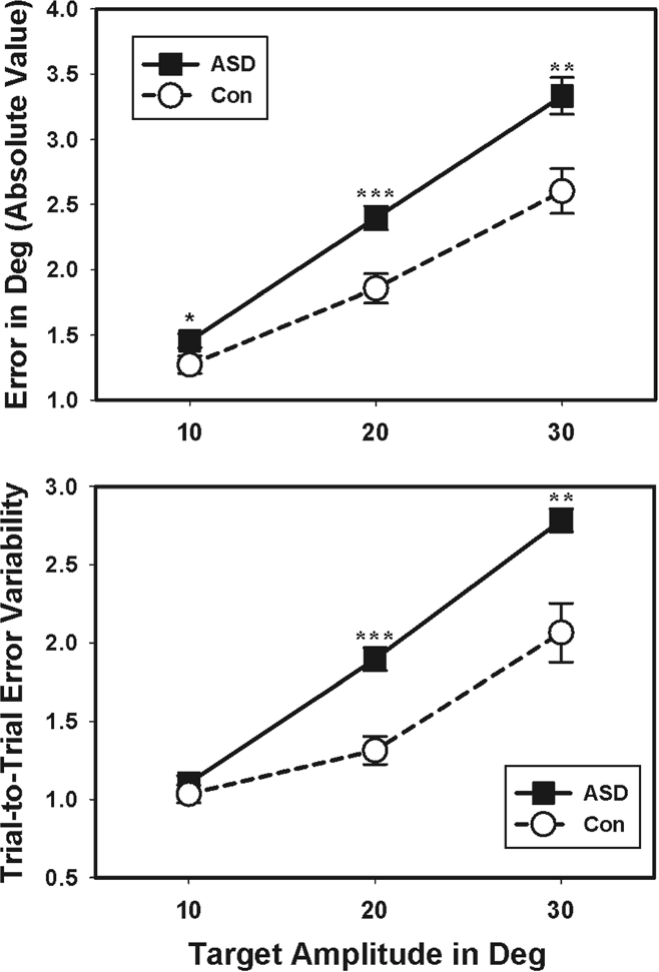
**Individuals with ASD showed reduced accuracy of saccades compared to healthy controls.** Absolute error of saccades in degrees of visual angle was increased in individuals with ASD (top). Individuals with ASD also demonstrated increased trial-to-trial variability of saccade accuracy compared to controls (bottom). Both abnormalities were greater for larger saccades at 20 and 30 degrees compared to 10 degrees. *P <0.05; **P <0.01; ***P <0.001. ASD, autism spectrum disorder; Con, control; Deg, degrees.

Participants with ASD and healthy controls had similar mean amplitudes of primary saccades (F(1,104) = 0.47; *P* = 0.496), but trial-wise variability of primary saccade amplitudes was increased in ASD (F(1,103) = 12.44; *P* = 0.001), especially for larger saccades (F(2,102) = 15.31; *P* <0.001). Participants with ASD demonstrated an increase in the absolute error of their saccades, thus showing reduced accuracy (Table 2; Figure 3; F(1,104) = 17.40; *P* <0.001). This deficit was more severe at larger target step amplitudes (F(2,103) = 4.23; *P* = 0.024). ASD participants also showed greater trial-to-trial variability of saccade error (Figure 3; F(1,103) = 19.13; *P* <0.001), which was again more pronounced at larger target step amplitudes (F(2,102) = 5.17; *P* = 0.014). Thus, increased saccade variability reflected both saccade under-shooting and over-shooting rather than consistent hypo- or hypermetria.

### Number of corrective saccades

As the number of saccades made to acquire targets was non-normally distributed, non-parametric tests were used to test for group differences. Individuals with ASD and controls did not differ in the number of saccades made to acquire peripheral targets, regardless of the size of target displacement (Mann-Whitney U test = 1353; *P* = 0.780).

The number of saccades made to acquire the target was related to saccade error (ASD: Spearman’s rank correlation coefficient, or Spearman’s rho = 0.28; *P* = 0.025; control: rho = 0.48; *P* = 0.001) and error variability across trials (ASD: rho = 0.28; *P* = 0.026; control: rho = 0.35; *P* = 0.034) in both groups. Thus, as expected, participants with ASD and healthy controls with greater saccade error and error variability made more saccades to acquire peripheral targets.

### Saccade dynamics

**Figure 4:**
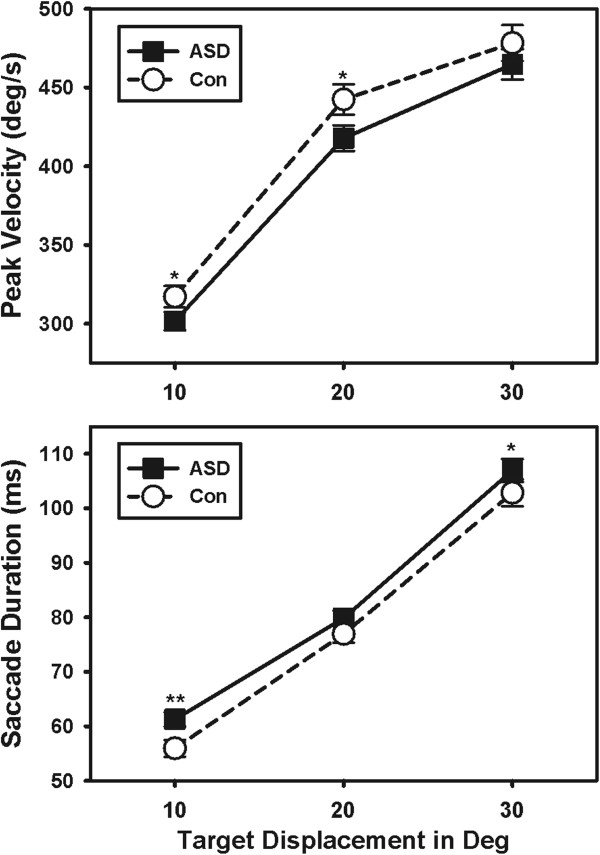
**Individuals with ASD showed reduced peak saccade velocities and increased saccade durations.** Individuals with ASD had reduced peak saccade velocities compared to healthy controls (top). The duration of their movements was increased compared to controls (bottom). *P <0.05; **P <0.01. ASD, autism spectrum disorder; Con, control; Deg, degrees.

Peak saccade velocities were reduced in ASD (Table 3; Figure 4; F(1,103) = 3.98; *P* = 0.049). This deficit was still evident after correcting velocity measures for saccade amplitudes (F(1,102) = 5.24; *P* = 0.024), thus, the velocity reduction did not result from smaller amplitude saccades in the patient group. Peak saccade velocity was not related to accuracy or variability of accuracy in either group (Additional files 1 and 2; *P*’s >0.077). The variability of peak velocities across trials was not different between groups (F(1,100) = 0.10; *P* = 0.754).

**Table 3:** **Saccade dynamics for participants with ASD and healthy controls**

	ASD	Control
**Peak velocity (deg/s)**		
10 deg	302 (46)*	317 (44)
20 deg	418 (64)*	442 (62)
30 deg	465 (77)	478 (74)
**Saccade duration (ms)**		
10 deg	61 (11)**	56 (10)
20 deg	80 (11)	77 (10)
30 deg	107 (17)*	103 (16)
**Peak acceleration (deg/s/s)**		
10 deg	20399 (615)	21850 (747)
20 deg	24025 (646)	25697 (785)
30 deg	25865 (695)	26746 (844)
**Duration of acceleration (ms)**		
10 deg	29.93 (5.85)**	27.26 (5.84)
20 deg	37.07 (6.14)*	35.01 (6.14)
30 deg	47.26 (8.40)*	43.46 (8.40)
**Peak deceleration (deg/s/s)**		
10 deg	17997 (561)	18849 (681)
20 deg	19756 (627)	20373 (762)
30 deg	19188 (674)	18383 (820)
**Duration of deceleration (ms)**		
10 deg	33.58 (5.58)	31.03 (5.58)
20 deg	45.46 (6.87)	45.02 (6.87)
30 deg	61.77 (11.01)	62.38 (11.01)

Mean (SDs). ASD, autism spectrum disorder; Deg, degrees; Deg/s, degrees per second; Ms, milliseconds; Deg/s/s, degrees per second per second.

*P <0.05; **P <0.01.

Saccade duration was increased in participants with ASD compared to controls (Figure 4; F(1,104) = 4.17; *P* = 0.044). The ratio of saccade duration:amplitude also was elevated in participants with ASD (F(1,104) = 8.11; *P* = 0.005), particularly at smaller target step amplitudes (F(2,100) = 4.38; *P* = 0.015). Saccade duration was not related to accuracy or variability of accuracy in either group (*P*’s >0.109). The trial-to-trial variability of saccade duration did not differ between groups (F(1,103) = 2.88; *P* = 0.093).

Although mean ratios of saccade velocity:amplitude and saccade duration:amplitude were significantly reduced and elevated in individuals with ASD, respectively, we found no group differences in the slopes of the saccade main sequence with either parameter (Additional file 3; *P*’s >0.429). Thus, individuals with ASD demonstrated abnormalities in velocity and duration that were consistent across saccade amplitudes.

### Acceleration and deceleration of saccades

Individuals with ASD and healthy controls had similar peak saccade accelerations (F(1,102) = 0.06; *P* = 0.804) and decelerations (F(1,102) = 0.2.02; *P* = 0.158). There was also no difference in trial-wise variability of peak acceleration (F(1,99) <0.01; *P* = 0.985) or peak deceleration (F(1,100) = 1.91; *P* = 0.170) across groups.

**Figure 5:**
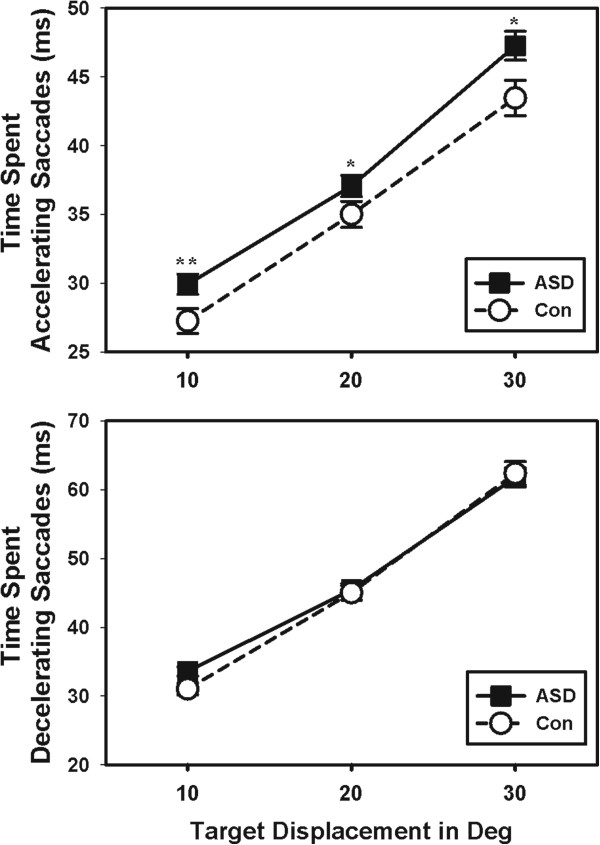
**Individuals with ASD took longer to reach peak velocity than healthy controls.** The time spent accelerating saccades was increased in ASD compared to healthy controls (top). Individuals with ASD showed similar durations of the deceleration phase of their saccades (bottom). * *P* <0.05; **P <0.01. ASD, autism spectrum disorder; Con, control; Deg, degrees.

However, individuals with ASD took more time to accelerate saccades to peak velocity than controls (Figure 5; F(1,104) = 6.35; *P* = 0.013). There was no group difference in the amount of time spent decelerating saccades (Figure 5; F(1,104) = 0.38; *P* = 0.541). Increased time spent accelerating saccades was not related to saccade accuracy (Additional file 1; r = -0.18; *P* = 0.354) or variability of accuracy (r = -0.06; *P* = 0.625) in ASD. However, increased time spent decelerating saccades was related to lower saccade error (r = -0.29; *P* = 0.021) and less variability of saccade error in ASD (r = -0.26; *P* = 0.039). Acceleration and deceleration durations were not related to accuracy measures in healthy controls (See Additional file 2). No group differences were found in the trial-wise variability of time spent accelerating (F(1,102 = 1.54; *P* = 0.217) or decelerating (F(1,102) = 2.30; *P* = 0.133) saccades.

Notably, impairments in saccade accuracy and saccade dynamics or the variability of these saccade measures were not significantly correlated in ASD (Additional files 1, 4 and 5). This suggests that different individuals with ASD express these two deficits separately, that alterations in brain systems regulating one saccade parameter is not the cause of dysfunction in the other, and that different brain systems are implicated by the two sets of abnormalities.

### Age-related changes in saccade accuracy and dynamics

Age-related reductions in saccade error (F(2,100) = 5.72; *P* = 0.004), saccade error variability (F(2,99) = 7.769; *P* = 0.001) and saccade amplitude variability (F(2,99) = 5.484; *P* = 0.006) were observed. Saccade latency (F(2,100) = 11.794; *P* <0.001) and saccade latency variability across trials (F(2,99) = 15.634; *P* <0.001) were reduced with increasing age of participants.. None of the age-related improvements in saccade performance differed between participant groups (Additional files 6, 7, 8 and 9; *P*’s >0.384), suggesting that deficits of patients relative to age-matched peers were relatively consistent across the age range studied. Saccade dynamics (such as velocity and duration) demonstrated no developmental change in either group (*P*’s >0.176).

### Clinical correlations

Saccade variables were not associated with intellectual functioning in either group (*P*’s >0.081). In participants with ASD, no saccade variable was significantly associated with clinical ratings of ASD features on the ADI-R or ADOS (*P*’s >0.177).

## Discussion

The present study examined visually-guided saccades to assess the functional integrity of cerebellar and brainstem sensorimotor systems and visual attention systems in ASD. Analyses revealed five key findings. First, participants with ASD demonstrated decreased accuracy of saccadic eye movements, and the accuracy of their saccades was more variable across trials. The increased variability in performance suggests a deficit in the cerebellar variability-reducing functions, leading to a reduced capacity for making compensatory adjustments to ensure consistent and accurate saccade execution. This replicates earlier findings [9, 13–16] indicating functional disturbances within cerebellar systems controlling the accuracy and consistency of eye movement trajectories, an interpretation that is supported by recent observations of saccade adaptation abnormalities in ASD [47, 48]. Second, peak saccade velocities were lower and durations were longer in participants with ASD. These abnormalities in peak velocity and duration suggest reduced pontine brainstem burst cell excitatory activity and/or increased omnipause cell inhibitory activity that alter the metrics of saccadic eye movements. These deficits were consistent across saccade amplitudes.

Third, patients spent more time accelerating saccades to reach peak velocity but not decelerating them. This abnormal eye movement activity has not been reported previously in ASD. It suggests poor temporal synchronization of burst cell and omnipause cell activity within pontine nuclei, with excessive inhibition or reduced excitatory drive in the early phase of saccade execution. A similar pattern of a protracted acceleration phase during finger movements has also been reported in ASD [72], thus abnormal input to multiple motor systems from the cerebellum could contribute to movement acceleration deficits across effector systems. Fourth, age-related improvements in saccade performance were similar across patient and control groups, suggesting a similar developmental profile of oculomotor function in ASD as in neurotypical individuals across the age span studied. Last, participants with ASD were not differentially impacted by gap and overlap conditions relative to controls, failing to provide evidence for an alteration in exogenous visual attentional systems in ASD. Mean response latencies also were unimpaired. Both of these observations are consistent with prior reports that automatic visual orienting attention systems are not impaired in ASD [9, 13, 14, 73]. However, increased latency variability in participants with ASD does indicate increased variability in attentional control.

Our characterization of saccade deficits in ASD is more analogous to patterns seen in patients with ataxia than other common neurodevelopmental or neuropsychiatric disorders [67, 74]. Individuals with spinocerebellar ataxia [75] and Friederich’s ataxia [76] show a similar pattern of reduced velocity, increased duration and dysmetria as seen in our patients. Notably, however, prolonged saccade duration in these samples occurs in the deceleration phase, and so the pattern we observed is a distinct one. Evidence of impaired saccade accuracy has been reported in Reading Disorder [77] and Tourette’s syndrome [78], but neither patient sample has been shown to have alternations in saccade dynamics. Thus, our findings of saccade deficits in ASD seem more indicative of a fundamental motor disturbance, rather than a problem of higher neurocognitive control.

### Saccade accuracy

Consistent with previous reports by our group and others [9, 13–15], we demonstrated saccade dysmetria in ASD. Four findings regarding our observation of reduced saccade accuracy are noteworthy. First, participants with ASD showed greater trial-to-trial variability in the accuracy of their saccades, indicating that increased variability in motor precision is present not only across affected individuals, but also within individual patients. Second, participants with ASD showed more pronounced dysmetria when making larger saccades, indicating that patients become more impaired when demands on the motor system are increased, a finding previously found by our group and others [13, 17, 18].

Third, despite the increased saccade amplitude error, we found no evidence of reduced saccade amplitudes, in contrast to prior reports of hypometria in ASD [9, 13–15]. Instead, our data demonstrated both hypometric and hypermetric saccades in ASD, with average saccade amplitude similar to that of healthy controls. The larger range of saccade amplitudes assessed and the use of multiple peripheral targets in the current study may contribute to the difference in our findings relative to previous studies. Fourth, despite an increase in error variability in ASD, the patient group did not demonstrate an increase in trial-to-trial variability of the duration of saccade deceleration, reflecting a failure to use online feedback processes to reduce saccade error.

One mechanism whereby the cerebellum controls movement accuracy is via online adjustments to the duration of eye movements, which occurs primarily during the deceleration phase of the saccade [31]. Cerebellar projections originating in the oculomotor vermis and relayed via caudal fastigial nuclei innervate pontine burst and omnipause neurons to modulate saccade durations and amplitudes [29, 79]. Thus, input from the cerebellum to the brainstem can reduce saccade error by providing a command to extend or reduce the duration of dysmetric movements so they end closer to the target location [25, 31, 33, 80, 81]. The observations that the trial-wise variability of saccade deceleration duration were not increased in ASD despite the increased variability in saccade accuracy suggests a reduced ability to either estimate expected error during saccade execution or to use those estimations to modify saccades in transit to reduce their spatial error.

Our findings thus indicate a deficit in the variability-reducing function of the cerebellum in ASD, resulting in both hypometric and hypermetric saccades. Purkinje cells of the dorsal cerebellar vermis are the sole output of the cerebellar cortex, and these cells are known to play a major role in controlling endpoint accuracy of saccades [33, 82, 83]. Selective damage to the oculomotor vermis within the cerebellum (lobules VI to VII) produces increased trial-to-trial variability of saccades in both non-human primates and in clinical studies, similar to findings observed in the present study [27–29, 31, 84]. This neurobehavioral evidence of impaired variability-reducing function in the cerebellum is consistent with postmortem studies documenting cerebellar histopathology in ASD, including reduced size and density of Purkinje cells [39, 42, 44–46].

### Saccade dynamics

One possible explanation for the altered saccade velocity and duration, and the extended acceleration but not deceleration phase of the saccade in our ASD participants, is an imbalance of excitatory and inhibitory signaling in the pontine brainstem. Duration of activity in pontine burst neurons is tightly correlated with saccade duration [85] and likewise, firing rates of the burst neurons are highly related to peak saccade velocity [86]. Omnipause cells tonically inhibit burst cells prior to a saccadic eye movement, and then become active again to terminate the movement. The increased time to accelerate saccades thus implicates enhanced inhibitory activity of pontine omnipause cells, diminished excitatory activity of burst cells or a reduced ability to synchronize the onset and offset of excitatory and inhibitory signaling. Our observation of altered saccade accelerations is consistent with prior anatomical studies of individuals with ASD documenting hypoplasia and cellular pathology within pontine nuclei [34, 35]. Although the brainstem is implicated by our data and prior ASD research, future studies are needed to confirm that the observed eye movement alternations directly result from brainstem abnormalities.

Alternatively, changes in altered saccade dynamics might result from abnormal upstream input to the pons from the superior colliculus, basal ganglia or cortical eye fields. Superior colliculus activity is known to influence saccade duration, peak velocity and acceleration [87–90], and also sends indirect input to pontine circuitry via the central mesencephalic reticular formation (cMRF) [24]. cMRF firing patterns regulate omnipause firing activity at the beginning and end of saccades [90]. Thus, it is possible that altered omnipause and burst cell firing rates may be a downstream effect of impairment within the superior colliculus, in its input or in the pathways between superior colliculus and excitatory and inhibitory pontine cells. While involvement of cortical eye fields and basal ganglia in these saccade dynamic abnormalities is possible, dysfunction in either region would be expected to have led to atypical gap/overlap effects, which were not seen in our study [24].

Another possibility is that the prolonged acceleration of eye movements could result from cerebellar dysfunction given the dense afferent and efferent projections between the cerebellum and pontine nuclei [29, 79, 91, 92]. Greater feedforward drive from the cerebellum to pons might extend saccade acceleration, but in this case similar alterations during the deceleration phase would be expected and these were not presently seen. Also, the independence of saccade accuracy and all movement dynamics provides evidence for a dissociation of abnormalities in the cerebellum and brainstem systems in ASD, which indicates that abnormal cerebellar input is not likely to be the primary cause of pontine dysfunction.

### Developmental improvements

Age-related improvements of saccade accuracy, saccade accuracy variability, saccade latency and saccade latency variability are consistent with prior findings in healthy control populations [21, 23, 71]. These findings are consistent with previous literature showing similar developmental trajectories of saccade latency [14, 93] and latency variability [93] in ASD and healthy control individuals across childhood and adolescence. Despite abnormalities of saccade accuracy and accuracy variability in ASD, individuals with ASD appear to benefit from developmental processes with a trajectory similar to that of healthy controls, at least in regard to the sensorimotor processes and age range examined in this study. Thus, these saccade abnormalities appear to reflect deficits emerging in childhood, if not earlier, that persist into adulthood. One implication of this pattern is that these deficits may be a useful biomarker for early detection but studies tracking these impairments to the first years of life are needed to characterize their onset and course.

### Clinical relationships

We observed no significant relationships between saccade measures and clinical features of ASD. Oculomotor control is supported by cortico-cerebellar-brainstem circuits which are notably distinct from those believed relevant to the social communication deficits and restricted, repetitive behaviors in ASD. Thus, even though sensorimotor deficits are present in the majority of individuals with ASD, they may represent additional neural system dysfunctions in ASD bearing limited relationship to those impacting neural substrates related to the disorder’s core diagnostic features. Furthermore, although we did not find evidence of a relationship between oculomotor dysfunctions and clinically-rated social communication abnormalities in ASD, it is possible that the observed alterations in saccade accuracy and dynamics are related to the ability to coordinate gaze during social interactions or when processing faces, as suggested by previous eye-tracking studies [6, 7]. Reduced ability to consistently modulate saccadic eye movement amplitudes could negatively impact early social experiences, which could have downstream effects on social and cognitive developments [8, 94].

## Conclusions

This study provides novel evidence that the accuracy and dynamics of visually-guided saccades are abnormal in ASD, implicating both cerebellum and brainstem systems in this disorder. Our findings are distinct from those that have been reported in other common neurodevelopmental and neuropsychiatric disorders, suggesting that these deficits may be unique to ASD, and provide biomarkers and/or endophenotypes for future research into this heterogeneous disorder. Our neurophysiological and functional observations are consistent with previous anatomic evidence from *in vivo* morphometric and postmortem studies that have demonstrated abnormalities in cerebellar and brainstem regions. Compromised cerebellar and brainstem function suggest that neural systems other than those related to key diagnostic features of ASD are affected in this disorder. These may lead to sensorimotor coordination impairments which impact on multiple aspects of day-to-day functioning [95].

## Electronic supplementary material

Additional file 1: Table S1: Relationships between saccade parameters for participants with ASD. Correlations of primary saccade parameters for participants with ASD. (DOCX 13 KB)

Additional file 2: Table S2: Relationships between mean saccade parameters for healthy controls. Correlations of primary saccade parameters for healthy control participants. (DOCX 11 KB)

Additional file 3: Figure S1: Main sequence relationships between peak saccade velocity and amplitude (top) and saccade duration and amplitude (bottom). Participants with ASD and healthy controls demonstrated similar slopes of the saccade main sequence for both parameters. Abnormalities observed in saccade velocity and duration thus appear to be consistent across saccade amplitudes in ASD. (TIFF 5 MB)

Additional file 4: Table S3: Relationships between saccade performance variability (SD) across variables for participants with ASD. Correlations of the variability of primary saccade parameters for participants with ASD. (DOCX 11 KB)

Additional file 5: Table S4: Relationships between saccade performance variability (SD) across variables for healthy controls. Correlations of the variability of primary saccade parameters for healthy control participants. (DOCX 11 KB)

Additional file 6: Table S5: Saccade accuracy and latencies during gap trials for participants with ASD and healthy controls. Saccade accuracy and latency variables during gap trials are presented for each participant group and age group. (DOCX 16 KB)

Additional file 7: Table S6: Saccade accuracy and latencies during overlap trials for participants with ASD and healthy controls. Saccade accuracy and latency variables during overlap trials are presented for each participant group and age group. (DOCX 16 KB)

Additional file 8: Table S7: Saccade dynamics during gap trials for participants with ASD and healthy controls. Saccade dynamic variables during gap trials are presented for each participant group and age group. (DOCX 16 KB)

Additional file 9: Table S8: Saccade dynamics during overlap trials for participants with ASD and healthy controls. Saccade dynamic variables during overlap trials are presented for each participant group and age group. (DOCX 16 KB)

## Abbreviations

ADI-R: Autism Diagnostic Interview-Revised
ADOS: Autism Diagnostic Observation Schedule
ASD: Autism spectrum disorder
cMRG: central mesencephalic reticular formation
EOG: Electro-oculography
IQ: Intelligence quotient
MRI: Magnetic resonance imaging
PDD-NOS: Pervasive Development Disorder-Not Otherwise Specified.
