# Supplementary material for: Saccadic eye movement abnormalities in autism spectrum disorder indicate dysfunctions in cerebellum and brainstem
Source: Mol Autism. 2014 Sep 16;5:47. doi: 10.1186/2040-2392-5-47 (PMC4233053; doi:10.1186/2040-2392-5-47)
Supplement: Supplementary file 1 — Additional file 1: Table S1: Relationships between saccade parameters for participants with ASD. Correlations of primary saccade parameters for participants with ASD. (DOCX 13 KB) [file 13229_2014_144_MOESM1_ESM.docx]

**Additional file 1: Table S1. Relationships between saccade parameters for participants with ASD.**

|  | Accuracy Variability | Velocity | Duration | Acceleration Duration | Deceleration Duration |
| --- | --- | --- | --- | --- | --- |
| Accuracy | .87*** | -.16 | -.22 | -.18 | -.29* |
| Accuracy Variability |  | -.17 | -.18 | -.06 | -.26* |
| Peak Velocity |  |  | -.65*** | -.46*** | -.65*** |
| Duration |  |  |  | .82*** | .86*** |
| Acceleration Duration |  |  |  |  | .44*** |

* p < 0.05; ** p < 0.01; *** p < 0.001.
