# Supplementary material for: Saccadic eye movement abnormalities in autism spectrum disorder indicate dysfunctions in cerebellum and brainstem
Source: Mol Autism. 2014 Sep 16;5:47. doi: 10.1186/2040-2392-5-47 (PMC4233053; doi:10.1186/2040-2392-5-47)
Supplement: Supplementary file 2 — Additional file 2: Table S2: Relationships between mean saccade parameters for healthy controls. Correlations of primary saccade parameters for healthy control participants. (DOCX 11 KB) [file 13229_2014_144_MOESM2_ESM.docx]

**Additional file 2: Table S2. Relationships between mean saccade parameters for healthy controls.**

|  | Accuracy Variability | Velocity | Duration | Acceleration Duration | Deceleration Duration |
| --- | --- | --- | --- | --- | --- |
| Accuracy | .86*** | -.25 | .09 | .21 | .01 |
| Accuracy Variability |  | -.30* | .17 | .21 | .14 |
| Peak Velocity |  |  | -.80*** | -.53*** | -.73*** |
| Duration |  |  |  | .82*** | .90*** |
| Acceleration Duration |  |  |  |  | .58*** |

* p < 0.05; ** p < 0.01; *** p < 0.001.
