# Supplementary figures and images for: Saccadic eye movement abnormalities in autism spectrum disorder indicate dysfunctions in cerebellum and brainstem
Source: Mol Autism. 2014 Sep 16;5:47. doi: 10.1186/2040-2392-5-47 (PMC4233053; doi:10.1186/2040-2392-5-47)

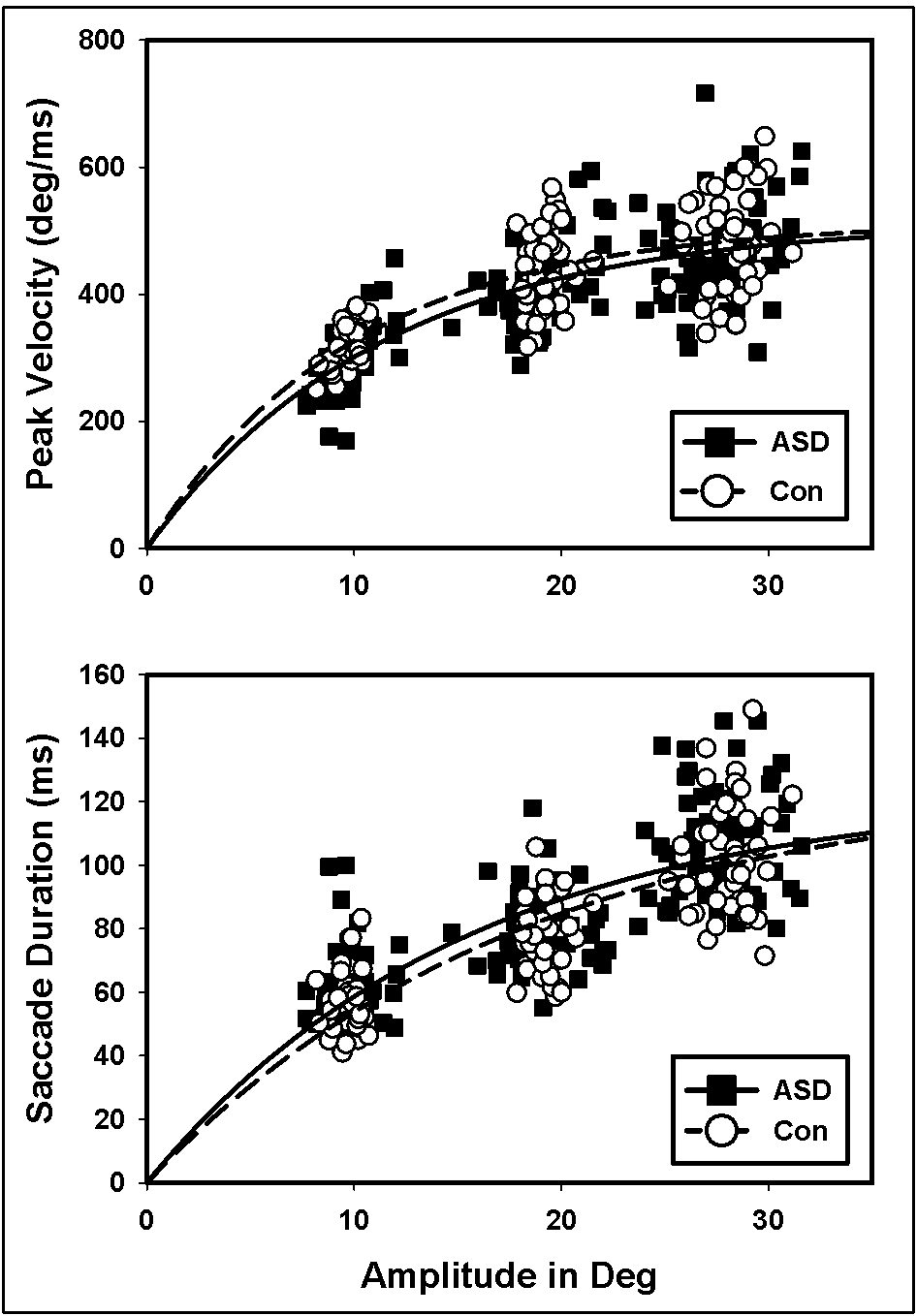

Supplement: Supplementary file 3 — Additional file 3: Figure S1: Main sequence relationships between peak saccade velocity and amplitude (top) and saccade duration and amplitude (bottom). Participants with ASD and healthy controls demonstrated similar slopes of the saccade main sequence for both parameters. Abnormalities observed in saccade velocity and duration thus appear to be consistent across saccade amplitudes in ASD. (TIFF 5 MB) [file 13229_2014_144_MOESM3_ESM.tiff]
