# Supplementary material for: Saccadic eye movement abnormalities in autism spectrum disorder indicate dysfunctions in cerebellum and brainstem
Source: Mol Autism. 2014 Sep 16;5:47. doi: 10.1186/2040-2392-5-47 (PMC4233053; doi:10.1186/2040-2392-5-47)
Supplement: Supplementary file 4 — Additional file 4: Table S3: Relationships between saccade performance variability (SD) across variables for participants with ASD. Correlations of the variability of primary saccade parameters for participants with ASD. (DOCX 11 KB) [file 13229_2014_144_MOESM4_ESM.docx]

**Additional file 4: Table S3. Relationships between saccade performance variability (SD) across variables for participants with ASD.**

|  | Velocity (SD) | Duration (SD) | Acceleration Duration (SD) | Deceleration Duration (SD) | Acceleration: Deceleration (SD) |
| --- | --- | --- | --- | --- | --- |
| Accuracy (SD) | .24 | .08 | .07 | .07 | .10 |
| Velocity (SD) |  | .27* | .32** | .24 | .28* |
| Duration (SD) |  |  | .89*** | .84*** | .23 |
| Acceleration Duration (SD) |  |  |  | .73*** | .32* |
| Deceleration Duration (SD) |  |  |  |  | .30* |

* p < 0.05; ** p < 0.01; *** p < 0.001.
