# Supplementary material for: Saccadic eye movement abnormalities in autism spectrum disorder indicate dysfunctions in cerebellum and brainstem
Source: Mol Autism. 2014 Sep 16;5:47. doi: 10.1186/2040-2392-5-47 (PMC4233053; doi:10.1186/2040-2392-5-47)
Supplement: Supplementary file 7 — Additional file 7: Table S6: Saccade accuracy and latencies during overlap trials for participants with ASD and healthy controls. Saccade accuracy and latency variables during overlap trials are presented for each participant group and age group. (DOCX 16 KB) [file 13229_2014_144_MOESM7_ESM.docx]

**Additional file 7: Table S6. Saccade accuracy and latencies during overlap trials for participants with ASD and healthy controls**

|  | ASD | | | CON | | |
| --- | --- | --- | --- | --- | --- | --- |
|  | 6-11 | 12-18 | 19+ | 6-11 | 12-18 | 19+ |
| **Saccade error (absolute value in deg of visual angle)** | | | | | | |
| 10 deg | 1.56 (.48) | 1.42 (.63) | 1.41 (.56) | 1.35 (.50) | 1.11 (.67) | 1.19 (.53) |
| 20 deg | 2.47 (.75) | 3.02 (.98) | 1.68 (.87) | 1.71 (.77) | 2.06 (1.03) | 1.74 (.81) |
| 30 deg | 3.43 (1.19) | 3.17 (1.6) | 2.54 (1.39) | 2.56 (1.23) | 2.45 (1.65) | 2.09 (1.30) |
| **Trial-wise variability (SD) of saccade error (absolute value in deg)** | | | | | | |
| 10 deg | 1.00 (.44) | 1.11 (.58) | 1.17 (.52) | 1.11 (.45) | 1.05 (.61) | 1.10 (.48) |
| 20 deg | 2.22 (.70) | 2.07 (.93) | 1.40 (.82) | 1.22 (.73) | 1.67 (.98) | 1.31 (.77) |
| 30 deg | 2.94 (1.48) | 2.89 (1.97) | 2.02 (1.74) | 2.28 (1.54) | 2.32 (2.07) | 1.39 (1.63) |
| **Saccade amplitude in deg** | | | | | | |
| 10 deg | 9.66 (.93) | 9.63 (1.21) | 10.01 (1.08) | 9.62 (.95) | 10.09 (1.28) | 9.56 (1.01) |
| 20 deg | 19.32 (1.28) | 18.50 (1.68) | 19.17 (1.49) | 19.68 (1.31) | 19.51 (1.77) | 19.19 (1.39) |
| 30 deg | 28.03 (1.85) | 27.99 (2.91) | 28.36 (2.16) | 28.24 (1.91) | 28.49 (2.56) | 28.75 (2.02) |
| **Trial-wise variability (SD) of saccade amplitude in deg** | | | | | | |
| 10 deg | 1.96 (.67) | 1.81 (.89) | 1.83 (.79) | 2.13 (.70) | 1.74 (.94) | 1.70 (.74) |
| 20 deg | 3.01 (.92) | 2.93 (1.21) | 2.21 (1.08) | 2.20 (.95) | 2.70 (1.28) | 2.30 (1.01) |
| 30 deg | 4.00 (1.37) | 4.42 (1.81) | 3.23 (1.61) | 3.03 (1.43) | 3.11 (1.91) | 2.51 (1.51) |
| **Saccade latency in ms** | | | | | | |
| 10 deg | 334 (54) | 268 (72) | 267 (64) | 332 (56) | 267 (76) | 283 (60) |
| 20 deg | 363 (57) | 278 (75) | 281 (67) | 375 (59) | 296 (79) | 299 (63) |
| 30 deg | 397 (62) | 350 (81) | 328 (72) | 403 (64) | 333 (86) | 343 (68) |
| **Trial-wise within-subject variability (SD) of saccade latency in ms** | | | | | | |
| 10 deg | 102 (33) | 85 (44) | 81 (39) | 97 (34) | 77 (46) | 57 (36) |
| 20 deg | 116 (35) | 94 (47) | 81 (41) | 100 (37) | 62 (49) | 55 (39) |
| 30 deg | 104 (34) | 99 (44) | 80 (39) | 94 (35) | 104 (47) | 80 (37) |
