# Supplementary material for: Saccadic eye movement abnormalities in autism spectrum disorder indicate dysfunctions in cerebellum and brainstem
Source: Mol Autism. 2014 Sep 16;5:47. doi: 10.1186/2040-2392-5-47 (PMC4233053; doi:10.1186/2040-2392-5-47)
Supplement: Supplementary file 8 — Additional file 8: Table S7: Saccade dynamics during gap trials for participants with ASD and healthy controls. Saccade dynamic variables during gap trials are presented for each participant group and age group. (DOCX 16 KB) [file 13229_2014_144_MOESM8_ESM.docx]

**Additional file 8: Table S7. Saccade dynamics during gap trials for participants with ASD and healthy controls**

|  | ASD | | | CON | | |
| --- | --- | --- | --- | --- | --- | --- |
|  | 6-11 | 12-18 | 19+ | 6-11 | 12-18 | 19+ |
| **Peak velocity (deg/s)** | | | | | | |
| 10 deg | 291 (48) | 298 (47) | 325 (50) | 305 (48) | 315 (50) | 335 (47) |
| 20 deg | 419 (72) | 421 (71) | 444 (75) | 434 (72) | 447 (65) | 442 (70) |
| 30 deg | 446 (82) | 464 (80) | 481 (84) | 480 (82) | 467 (85) | 495 (79) |
| **Saccade duration (ms)** | | | | | | |
| 10 deg | 63 (11) | 61 (11) | 61 (11) | 55 (11) | 55 (12) | 52 (11) |
| 20 deg | 79 (11) | 79 (10) | 76 (11) | 77 (11) | 79 (11) | 73 (10) |
| 30 deg | 104 (18) | 105 (18) | 109 (18) | 100 (18) | 107 (19) | 98 (17) |
| **Peak acceleration (deg/s/s)** | | | | | | |
| 10 deg | 18394 (5370) | 20642 (5247) | 23395 (5474) | 20291 (5370) | 20495 (5609) | 24895 (5200) |
| 20 deg | 24280 (6890) | 25586 (6732) | 25658 (7116) | 24402 (6890) | 25893 (7197) | 26803 (6671) |
| 30 deg | 24762 (6597) | 26597 (6445) | 29755 (6813) | 26190 (6597) | 25537 (6890) | 28313 (6387) |
| **Duration of acceleration (ms)** | | | | | | |
| 10 deg | 31.42 (6.30) | 29.91 (6.17) | 29.11 (6.51) | 29.94 (6.31) | 26.82 (6.59) | 24.78 (6.10) |
| 20 deg | 37.12 (6.52) | 36.64 (6.37) | 35.20 (6.73) | 34.28 (6.52) | 34.26 (6.81) | 33.41 (6.31) |
| 30 deg | 47.16 (10.11) | 45.45 (9.88) | 47.87 (10.44) | 43.78 (10.10) | 44.28 (10.56) | 43.60 (9.78) |
| **Peak deceleration (deg/s/s)** | | | | | | |
| 10 deg | 16865 (4547) | 17230 (4731) | 19224 (4696) | 17585 (4547) | 17450 (4749) | 20140 (4402) |
| 20 deg | 19444 (5677) | 20005 (4563) | 21771 (5863) | 19363 (5677) | 19799 (5929) | 20545 (5497) |
| 30 deg | 19392 (5778) | 18584 (5639) | 20858 (5968) | 18433 (5778) | 15756 (6035) | 20160 (5595) |
| **Duration of deceleration (ms)** | | | | | | |
| 10 deg | 34.28 (5.98) | 33.35 (5.85) | 35.31 (6.18) | 29.75 (5.98) | 30.04 (6.25) | 29.61 (5.79) |
| 20 deg | 45.15 (7.17) | 44.24 (7.02) | 43.82 (7.41) | 45.09 (7.17) | 47.64 (7.49) | 42.48 (6.94) |
| 30 deg | 60.80 (11.71) | 60.96 (11.45) | 63.75 (12.09) | 59.14 (11.71) | 69.24 (12.23) | 58.73 (11.34) |
