# Supplementary material for: Saccadic eye movement abnormalities in autism spectrum disorder indicate dysfunctions in cerebellum and brainstem
Source: Mol Autism. 2014 Sep 16;5:47. doi: 10.1186/2040-2392-5-47 (PMC4233053; doi:10.1186/2040-2392-5-47)
Supplement: Supplementary file 9 — Additional file 9: Table S8: Saccade dynamics during overlap trials for participants with ASD and healthy controls. Saccade dynamic variables during overlap trials are presented for each participant group and age group. (DOCX 16 KB) [file 13229_2014_144_MOESM9_ESM.docx]

**Additional file 9: Table S8. Saccade dynamics during OVERLAP trials for participants with ASD and healthy controls**

|  | ASD | | | CON | | |
| --- | --- | --- | --- | --- | --- | --- |
|  | 6-11 | 12-18 | 19+ | 6-11 | 12-18 | 19+ |
| **Peak velocity (deg/s)** | | | | | | |
| 10 deg | 300 (48) | 290 (61) | 318 (56) | 324 (49) | 333 (66) | 318 (52) |
| 20 deg | 403 (63) | 401 (77) | 434 (70) | 451 (62) | 448 (83) | 452 (66) |
| 30 deg | 470 (73) | 442 (92) | 495 (84) | 480 (74 | 492 (99) | 489 (77) |
| **Saccade duration (ms)** | | | | | | |
| 10 deg | 62 (12) | 56 (15) | 63 (14) | 61 (12) | 57 (17) | 56 (13) |
| 20 deg | 80 (13) | 82 (17) | 84 (15) | 80 (13) | 79 (18) | 76 (14) |
| 30 deg | 105 (16) | 113 (21) | 110 (19) | 103 (17) | 101 (22) | 102 (18) |
| **Peak acceleration (deg/s/s)** | | | | | | |
| 10 deg | 19381 (5271) | 20736 (5150) | 22555 (5444) | 20011 (5271) | 22064 (5506) | 22774 (5104) |
| 20 deg | 22605 (6047) | 24169 (5910) | 26285 (6245) | 25585 (6047) | 24957 (6316) | 26285 (5855) |
| 30 deg | 25522 (6056) | 25417 (5917) | 27097 (6254) | 26204 (6056) | 27533 (6325) | 26500 (5863) |
| **Duration of acceleration (ms)** | | | | | | |
| 10 deg | 31.21 (6.34) | 26.42 (8.00) | 29.49 (7.26) | 30.99 (6.42) | 26.82 (8.62) | 26.35 (6.81) |
| 20 deg | 36.75 (7.48) | 38.92 (9.43) | 38.13 (8.56) | 36.56 (7.57) | 35.99 (10.16) | 35.58 (8.03) |
| 30 deg | 46.88 (9.06) | 49.30 (11.44) | 48.09 (10.38) | 42.83 (9.18) | 39.58 (12.31) | 45.13 (9.73) |
| **Peak deceleration (deg/s/s)** | | | | | | |
| 10 deg | 17781 (5290) | 17267 (5162) | 19834 (5463) | 18608 (5290) | 19413 (5525) | 19541 (5122) |
| 20 deg | 18914 (5102) | 18558 (4979) | 21311 (5269) | 20075 (5102) | 19438 (5328) | 22462 (4940) |
| 30 deg | 19860 (5979) | 17793 (5835) | 193687 (6175) | 18606 (5979) | 16833 (6425) | 19223 (5789) |
| **Duration of deceleration (ms)** | | | | | | |
| 10 deg | 33.60 (7.57) | 32.40 (7.41) | 32.11 (7.82) | 31.95 (7.58) | 33.60 (7.19) | 31.70 (7.34) |
| 20 deg | 47.77 (8.19) | 47.09 (8.01) | 47.54 (8.46) | 45.51 (8.19) | 47.64 (8.56) | 43.39 (7.93) |
| 30 deg | 59.32 (11.70) | 63.82 (11.44) | 63.92 (1.32) | 61.12 (11.70) | 68.83 (12.21) | 61.12 (11.32) |
